# ApoE4 delays dendritic spine formation during neuron development and accelerates loss of mature spines *in vitro*

**DOI:** 10.1042/AN20130043

**Published:** 2014-01-13

**Authors:** Evelyn Nwabuisi-Heath, G. William Rebeck, Mary Jo LaDu, Chunjiang Yu

**Affiliations:** *Department of Anatomy and Cell Biology, University of Illinois at Chicago, Chicago, IL, U.S.A.; †Department of Neuroscience, Georgetown University, Washington, DC, U.S.A.

**Keywords:** Alzheimer’s disease, apolipoprotein E, dendritic spine, glutamate receptor, neuron development, synapse dysfunction, AD, Alzheimer’s disease, AMPAR, α-amino-3-hydroxy-5-methyl-4-isoxazolepropionic acid ionic glutamate receptor, apoE, apolipoprotein E, APOE-TR, APOE4-targeted replacement, DIV, day-in-vitro, NMDAR, *N*-methyl-D-aspartate glutamate receptor, wt, wild-type

## Abstract

The ε4 allele of the gene that encodes apolipoprotein E (APOE4) is the greatest genetic risk factor for Alzheimer's disease (AD), while APOE2 reduces AD risk, compared to APOE3. The mechanism(s) underlying the effects of APOE on AD pathology remains unclear. *In vivo*, dendritic spine density is lower in APOE4-targeted replacement (APOE-TR) mice compared with APOE2- and APOE3-TR mice. To investigate whether this apoE4-induced decrease in spine density results from alterations in the formation or the loss of dendritic spines, the effects of neuron age and apoE isoform on the total number and subclasses of spines were examined in long-term wild-type neurons co-cultured with glia from APOE2-, APOE3- and APOE4-TR mice. Dendritic spine density and maturation were evaluated by immunocytochemistry via the presence of drebrin (an actin-binding protein) with GluN1 (NMDA receptor subunit) and GluA2 (AMPA receptor subunit) clusters. ApoE isoform effects were analyzed via a method previously established that identifies phases of spine formation (day-in-vitro, DIV10–18), maintenance (DIV18–21) and loss (DIV21–26). In the formation phase, apoE4 delayed total spine formation. During the maintenance phase, the density of GluN1+GluA2 spines did not change with apoE2, while the density of these spines decreased with apoE4 compared to apoE3, primarily due to the loss of GluA2 in spines. During the loss phase, total spine density was lower in neurons with apoE4 compared to apoE3. Thus, apoE4 delays total spine formation and may induce early synaptic dysfunction via impaired regulation of GluA2 in spines.

## INTRODUCTION

Apolipoprotein E (apoE) is a protein component of plasma lipoproteins known primarily for its role in lipid transport and maintenance of cholesterol homoeostasis in the periphery and the central nervous system. Within the brain, apoE is synthesized primarily by glia, and secreted for subsequent interactions with members of the apoE family of receptors expressed by neurons and glia (Boyles et al., 1985; Pitas et al., 1987; Linton et al., 1991; Rapp et al., 2006). ApoE is encoded by the APOE gene with three common alleles in the human population: ε2, ε3 and ε4. Compared to APOE3, inheritance of APOE4 increases risk of Alzheimer's disease (AD) 4- to 15-fold depending on the presence of one or two alleles, and reduces the age of AD onset (Strittmatter and Roses, 1996). In contrast, APOE2 reduces AD risk 2- and 4-fold (Corder et al., 1994). The specific mechanisms underlying APOE-dependent AD risk remain unclear.

AD manifests as a loss of short-term memory, followed by loss of long-term memory and cognition. Evidence demonstrates loss of hippocampal synapses as a major structural correlate of the cognitive dysfunctions in AD (Scheff et al., 2006, 2007). ApoE plays a critical role in the regulation of neuron and synapse development, maintenance and repair (Valastro et al., 2001; Kim et al., 2011). These functions of apoE are modulated by apoE isoform and may serve as a source of the APOE-associated AD risk. Initial *in vitro* analyses of apoE isoform effects on neurons show that apoE3 enhances, while apoE4 inhibits or has no effect on, neurite extension during neuron development (Nathan et al., 1995; Sun et al., 1998; Nathan et al., 2002). In the cortex of human APOE-targeted replacement (APOE-TR) mice (Sullivan et al., 1997, 1998) dendritic spine density and dendritic complexity was lowest in APOE4-TR mice, compared with APOE2- and APOE3-TR mice (Dumanis et al., 2009). In hippocampal slice cultures from APOE-TR mice, synaptic plasticity was altered in the dentate gyrus and CA1 regions of APOE4-TR mice (Trommer et al., 2004; Korwek et al., 2009).

While apoE4 is associated with a reduction in the density of dendritic spines relative to apoE2 and apoE3 both *in vitro* and *in vivo* (Ji et al., 2003; Dumanis et al., 2009), it is unclear whether apoE4 inhibits the formation or enhances the loss of spines. Dendritic spines are highly dynamic structures that undergo cycles of extension and retraction, although as neurons mature more stable spines gradually replace motile spines. The dynamic nature of spines suggests that dendritic spine density reflects the net balance between spine formation and elimination. Longitudinal analysis of spine density demonstrates an initial increase in spine density, a relatively stable phase, followed by a decrease in spine density as neurons mature (Papa et al., 1995; Nwabuisi-Heath et al., 2012). Therefore, longitudinal analysis of spine density provides a model to evaluate the relative effects of apoE isoform on the formation and elimination of spines.

*N*-methyl-D-aspartate and α-amino-3-hydroxy-5-methyl-4-isoxazolepropionic acid ionic glutamate receptors (NMDAR and AMPAR, respectively) are the primary mediators of excitatory synapse transmission (Thal, 2012) and are critically involved in the formation, maturation and stability of dendritic spines (Petralia et al., 1999; Alvarez et al., 2007; Wirths and Bayer, 2012). Ultimately, memory and cognitive deficits result from synaptic dysfunction, which may be the result of glutamate receptor-dependent alterations in spines, as spines that lack NMDAR or AMPAR exhibit impaired or loss of synaptic transmission (Ye et al., 2000). Interestingly, apoE isoforms have been shown to differentially affect NMDAR and AMPAR signaling and trafficking: in reelin-stimulated cultures, apoE4 inhibits NMDAR subunit phosphorylation and surface expression of AMPAR, compared with apoE2 and apoE3 (Chen et al., 2010). Losses of NMDAR and AMPAR in cortical neurons with age have also been reported (Hof et al., 2002), however, apoE isoform effects on NMDAR and AMPAR loss remain unknown.

In the current study, the effects of apoE isoform and neuron age on the density and composition of NMDAR and AMPAR on dendritic spines were examined in long-term wild-type (wt) neurons co-cultured with glia from APOE-TR mice. These effects were evaluated during phases of dendritic spine formation (day-in-vitro, DIV10–18), maintenance (DIV18–21) and loss (DIV21–26) as a continuum, using a method previously designed for this *in vitro* model (Nwabuisi-Heath et al., 2012). This neuron–glia co-culture model was utilized to take advantage of the natively regulated secretion of apoE by glia isolated from APOE2-, APOE3- and APOE4-TR mice. The current study demonstrates that apoE4 delayed spine formation during neuron development, compared with apoE2 and apoE3. With increasing neuron age, apoE4 induced a loss of mature spines compared to apoE2 and apoE3, primarily via the loss of AMPAR from spines.

## MATERIALS AND METHODS

### Primary neuron, glia, and neuron–glia co-cultures

Animals were handled according to the Institutional Animal Care and Use Committee (IACUC) protocols at the University of Illinois at Chicago, and the National Institutes of Health Guide for the Care and Use of Laboratory Animals. Primary neuron and glial cultures were prepared as previously described (Nwabuisi-Heath et al., 2012). Briefly, five to seven E17 embryos or postnatal-day 2/3 (P2/3) pups were used for primary neuron or glial culture, respectively. For co-culture, DIV5 neurons prepared from C57BL6 (Charles River, Jackson Labs) mouse embryos on 12 mm- or 15 mm-diameter coverslips were transferred into six-well plates containing tertiary glial cells prepared from APOE2-, APOE3- or APOE4-TR mice. Two or three coverslips were placed in each well with the neurons facing the glia. Paraffin wax spotted on the surface of the coverslips prevented direct contact between neurons and glia. Cells were co-cultured in neurobasal media containing 1×B27 supplement and 1×Glutamax. Every 3–4 days, 10% of the media was changed.

### Immunocytochemistry

On DIV 10, 14, 18, 21 and 26, neurons on coverslips were rinsed in artificial cerebrospinal fluid (ACSF: 145 mM NaCl, 3 mM KCl, 1 mM CaCl_2_, 10 mM Hepes, 1 mM MgCl_2_, and 8 mM dextrose), fixed with ice-cold methanol for 15 min, and permeabilized with 1×phosphate-buffered saline containing 0.025% Triton X-100 detergent (1×PBS-TX) for 10 min. After blocking with 3% BSA in 1×PBS for 30 min, neurons were incubated for 1 h with an antibody mixture containing: rabbit anti-GluR2 (Millipore, 1:50 dilution) for AMPAR subunit 2 (GluA2); mouse anti-NR1 (Pharmingen, 1:50 dilution) for NMDAR subunit 1 (GluN1); pig anti-drebrin (Fitzgerald, 1:500 dilution); and anti-MAP2 (Abcam, 1:500 dilution). Neurons were washed and incubated for 30 min with a mixture of corresponding Alexa fluorophore-labelled secondary antibodies (Invitrogen, 1:500 dilution) containing: donkey anti-rabbit 750, donkey anti-mouse 647, donkey anti-guinea pig 488, and goat anti-chicken 594. Following three 5 min washes with 1×PBS-TX, coverslips were mounted on slides with Prolong Gold Antifade reagent with DAPI (Invitrogen). Mounting media were allowed to cure overnight.

### Image acquisition, dendrite sampling and quantitative analysis

Image acquisition, dendrite sampling and quantitative analyses were performed as previously described (Nwabuisi-Heath et al., 2012). Briefly, wide-field images of neurons were acquired using a Zeiss Axio Imager M1 fluorescence microscope equipped with a Zeiss AxioCam HRm camera and controlled with Axiovision version 4.7 software. Images were captured with a 63× 1.4 NA oil objective. Three to five positions per coverslip were randomly selected and a 5×6 mosaic of 63× images per position were captured at 2776×2080 pixel resolution. Each mosaic contained an average of 15–20 neurons. For dendrite sampling, spiny neurons with discrete dendrites were marked, as determined by immunoreactivity for MAP2 and drebrin. All channels were then turned off except for MAP2 and DAPI for dendrite sampling. Dendrite segments 20 μm in length, from two to three dendrites per neuron, were sampled 50 μm away from the cell body. Distance and length measurements were performed using the Axiovision software measurement tool. Acquired 20 μm dendrite images were discarded in cases where two or more dendrites were in close proximity such that overlap of spines was suspected. Images of sampled dendrites were exported as tiff images for quantitative analysis.

As previously described (Nwabuisi-Heath et al., 2012), drebrin, GluN1 and GluA2 cluster quantifications were performed with ImageJ NIH software using custom plugins. For co-localization analysis, juxtaposed (within 1 pixel distance) and overlapping clusters were measured as co-localized. All measurements were performed on a per 20 μm dendrite basis. An average ‘n’ of 50 dendrite segments per isoform per time point was used for spine analysis. Data shown are representative of two independent experiments and are expressed as means±S.E.M. Statistical analyses of apoE isoform effects was performed by one-way ANOVA and Tukey's post-hoc test, *P*<0.05 (GraphPad Prism 5); and significance between time points for a single isoform was analyzed by unpaired Student's *t* tests with equal variance, *P*<0.01.

### Identification of spine subtypes

**Figure 1:**
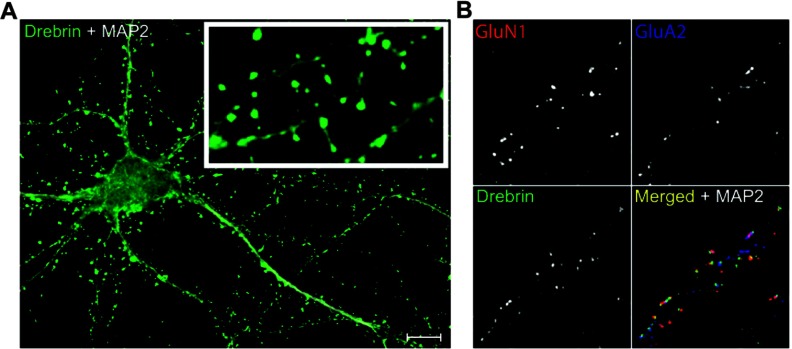
Drebrin, GluN1 and GluA2 clusters as markers for spines and spine subtypes (**A**) Representative image of DIV14 neuron showing drebrin clusters as spine markers. MAP2 was used as a dendrite marker. (**B**) Representative quadruple-stained dendrite segment showing debrin clusters co-localized with or without GluN1 or GluA2 clusters. In merged image: drebrin (green), GluN1 (red), GluA2 (blue), and MAP2 (white). Scale bar 10 μm.

Dendritic spines were identified using drebrin clusters as spine markers. Triple and double co-localizations of GluN1 and GluA2 clusters with drebrin clusters (Figure 1) were used to identify spine subtypes. Triply co-localized GluN1, GluA2 and drebrin clusters (GluN1+GluA2+drebrin clusters) identified mature spines. Immature spine densities were determined by subtracting the density of GluN1+GluA2+drebrin clusters from total drebrin clusters at each time point per isoform. The density of GluN1-only or GluA2-only spines at each time point per isoform was determined by subtracting GluN1+GluA2+drebrin clusters from the density of doubly co-localized GluN1+drebrin or GluA2+drebrin clusters, respectively. Drebrin-only spine densities were determined by subtracting the densities of GluN1+GluA2+drebrin, GluN1-only and GluA2-only clusters from the total drebrin cluster density.

## RESULTS

### ApoE isoform effects on total dendritic spine density

**Figure 2:**
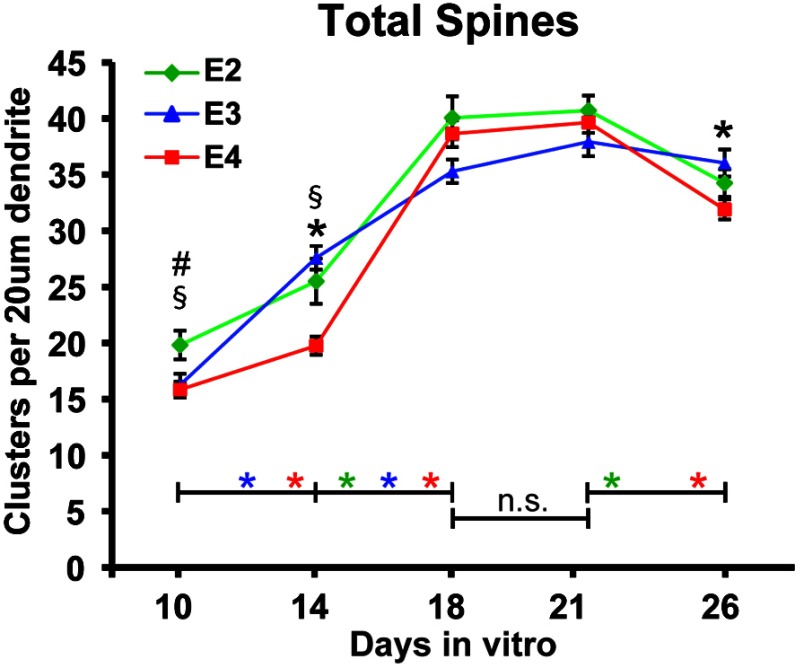
ApoE4 delays spine formation compared with apoE2 and apoE3 Density of drebrin clusters was quantified from 20 μm dendrite segments sampled 50 μm away from the cell body of DIV10–26 neurons grown with APOE2, APOE3 or APOE4 glia. # denotes significant differences at *P*<0.05, between E2 and E3, § between E2 and E4, and * between E3 and E4, one-way ANOVA, Tukey's post-hoc test. Along the *x*-axis, color matched * denotes significant differences between time points within apoE isoform using the Student's *t* test with equal variance, significance *P*<0.01; n.s., not significant. Values are expressed as means±S.E.M.

Dendritic spines have actin-rich, bulbous heads, distinguishing them from the more dynamic filopodia protrusions. Drebrin, an actin-binding protein, is enriched and clustered in spine heads. Therefore, drebrin clusters were used as markers for spines, to exclude contributions from filopodia extensions in spine density analyses (Figure 1). As previously reported, drebrin cluster density from DIV10 to DIV26 revealed phases of increasing spine density (spine formation phase, DIV 10–18), stabilization of spine density (spine maintenance phase, DIV 18–21), and decreasing spine density (spine loss phase, DIV 21–26) (Nwabuisi-Heath et al., 2012), in wt neurons co-cultured with glia isolated from APOE2-, APOE3- and APOE4-TR mice. For total spine density, the greatest density on DIV10 was with apoE2, with no difference between apoE3 and apoE4 (Figure 2). On DIV14, spine density was significantly lower with apoE4, with no difference between apoE2 and apoE3. Between DIV14 and DIV18, spine density increased in all three groups with no significant difference among the apoE isoforms. The lower density of spines with apoE4 early in development (DIV14), and the subsequent increase to levels comparable with apoE2 and apoE3 cultures (DIV18) suggest a delay, rather than a reduction, in spine formation with apoE4. From DIV18 to DIV21, total spine densities were comparable with apoE2, apoE3 and apoE4. However, by DIV26, significant decreases in spine density were observed with apoE2 and apoE4.

### ApoE isoform effects on density of dendritic spine subclasses

**Figure 3:**
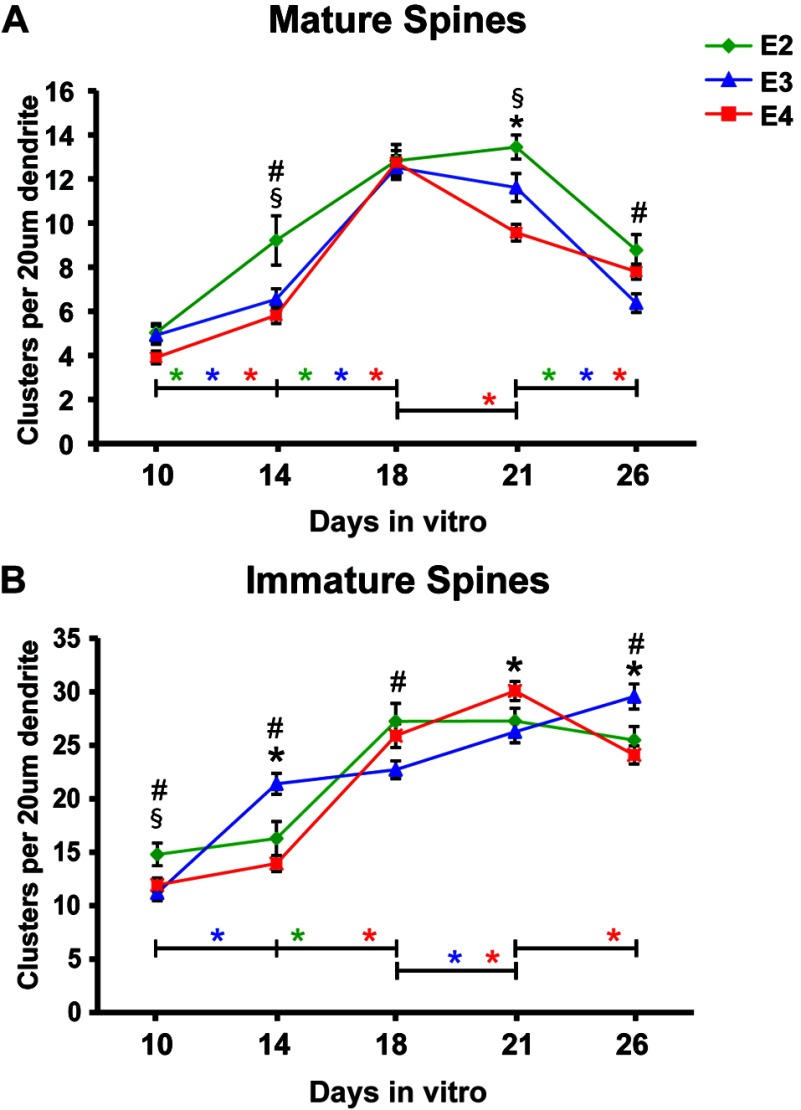
ApoE2 enhances the formation and maintenance of mature (GluN1+GluA2) dendritic spines, while ApoE4 accelerates loss of these spines (**A**) Density of triply co-localized GluN1, GluA2 and drebrin clusters was quantified from 20 μm dendrite segments sampled from DIV10 to DIV26 neurons grown with APOE2, APOE3 or APOE4 glia. (**B**) ‘Immature’ spine density was obtained by subtracting the density of triply co-localized GluN1, GluA2 and drebrin clusters from the density of drebrin clusters at each time point per apoE isoform. Significance differences defined as described for Figure 2.

Characteristics of mature functional dendritic spines include the presence of both NMDAR and AMPAR in the excitatory synapse on the spines. To determine if apoE isoform had specific effects on subpopulations of spines defined by the presence of these two types of glutamate receptors, we first analyzed the density of spines containing both the NMDAR subunit GluN1 and the AMPAR subunit GluA2 (Figure 3A and Table 1). On DIV10, the density of spines containing both NMDAR and AMPAR (GluN1+GluA2 positive) were comparable in all apoE groups. On DIV14, while the density of GluN1+GluA2 positive spines increased with all three apoE isoforms, apoE2 had a significantly greater effect than apoE3 and apoE4. On DIV18, these GluN1+GluA2 positive spines reached maximum density in all three isoforms with no differences. Thus, despite the delayed increase in total spine density with apoE4 in the formation phase, the density of apoE4 mature spines was comparable to apoE3, with apoE2 accelerated.

**Table 1:** ApoE isoform and neuron age effects on spine subclasses Values shown are density per 20 μm dendrite lengths, *n* equals average of 50 dendrite segments/isoform per time point. Data shown are representative of two independent experiments. # denotes significant differences at *P*<0.05, between E2 and E3, § between E2 and E4, and * between E3 and E4, one-way ANOVA and Tukey's post-hoc test. Values are means±S.E.M. Phases: formation, DIV10–18; maintenance, DIV18–21; and loss, DIV21–26

Type	DIV10	DIV14	DIV18	DIV21	DIV26
Mature spines					
GluN1+GluA2		# §		* §	#
ApoE2	5.0±0.4	9.2±1.1	12.8±0.7	13.4±0.5	8.8±0.7
ApoE3	4.9±0.4	6.5±0.5	12.5±0.5	11.6±0.6	6.4±0.4
ApoE4	3.9±0.3	5.8±0.4	12.7±0.5	9.6±0.4	7.8±0.3
Immature spine types					
GluN1-only	#	*	# *	*	*
ApoE2	6.0±0.7	7.6±1.0	15.5±1.3	15.4±0.7	13.7±0.8
ApoE3	4.1±0.4	8.5±0.6	12.1±0.6	14.0±0.6	15.9±0.8
ApoE4	4.8±0.4	5.4±0.4	14.7±0.7	16.5±0.6	12.1±0.6
GluA2-only		*	# *	# §	§
ApoE2	3.2±0.3	5.6±0.7	7.0±0.5	6.3±0.4	3.3±0.4
ApoE3	2.7±0.3	7.4±0.6	5.1±0.3	4.6±0.3	2.8±0.3
ApoE4	2.8±0.2	3.8±0.3	7.2±0.4	4.7±0.2	2.2±0.2
Drebrin-only				§	#
ApoE2	5.7±0.6	3.1±0.8	4.7±0.9	5.5±0.7	8.5±0.8
ApoE3	4.4±0.4	5.5±0.7	5.5±0.7	7.6±0.6	10.8±0.6
ApoE4	4.4±0.4	4.7±0.5	4.0±0.6	8.9±0.5	9.8±0.5

During the spine maintenance phase (DIV18–21), there was a significant decrease in the density of GluN1+GluA2-positive spines with only apoE4. Thus, by DIV21, the greatest density of GluN1+GluA2-positive spines was with apoE2, an intermediate density with apoE3 and the lowest density with apoE4. On DIV26, these GluN1+GluA2-positive spines decreased in all three groups to levels where densities were comparable between apoE2 and apoE4, and lowest with apoE3. Based on these data from DIV18–26, we conclude that apoE4 induces an early loss of functionally mature spines, compared with apoE2 and apoE3.

Next, we analyzed the effect of apoE isoform on immature spines, defined as spines that lack one or both of the glutamate receptor subunits, GluN1 or GluA2 (Figure 3B). On DIV10, immature spine density was greatest with apoE2, and comparable between apoE3 and apoE4. On DIV14, the highest spine density was with apoE3, with no significant increase with apoE2 and apoE4. This is the time point where the greatest density of mature spine was with apoE2 (Figure 3A). From DIV14 to DIV18, the density of immature spines increased with apoE2 and apoE4. On DIV21, further increases in density were observed with apoE3 and apoE4, but not with apoE2. At DIV26, immature spine densities plateaued for apoE2 and apoE3, but significantly decreased with apoE4. The increase in the density of immature spines at the maintenance phase (DIV18–21) suggests that the decrease in the density of mature spines may result from a loss of either GluN1 and/or GluA2.

**Figure 4:**
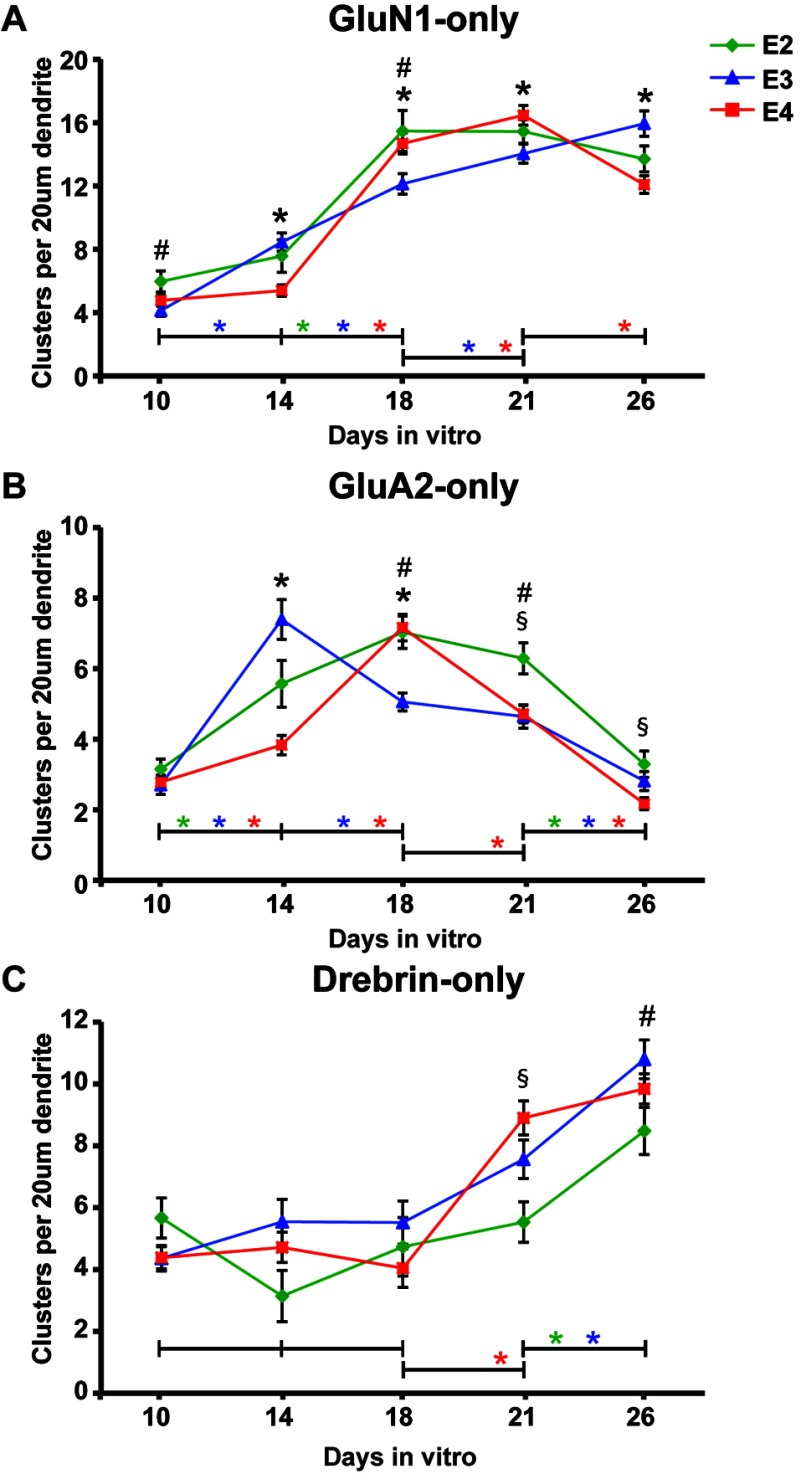
ApoE4 induces early loss of GluA2 from spines in mature neurons Densities of (**A**) GluN1-only, (**B**) GluA2-only and (**C**) drebrin-only spines from 20 μm dendrite segments sampled from DIV10 to DIV26 neurons grown with APOE2, APOE3 or APOE4 glia. Significance differences defined as described for Figure 2.

To determine the effect of apoE isoform on immature spine subclasses, analysis was separated to reflect changes in the densities of spines that express only GluN1 (GluN1-only), only GluA2 (GluA2-only), or neither GluN1 or GluA2 (drebrin-only) (Figure 4 and Table 1). On DIV10, GluN1-only spine density was greater with apoE2, and no difference with apoE4, compared to apoE3 (Figure 4A). On DIV14, GluN1-only spine density increased with apoE3, resulting in comparable densities between apoE2 and apoE3 and lower with apoE4. By DIV18, GluN1-only spines increased for all isoforms. Interestingly, the maximal density of GluN1-only spines with apoE4 was comparable to apoE2 and greater than apoE3. These data further support accelerated and delayed formation of new spines with apoE2 and apoE4, respectively. At the maintenance phase (DIV18–21), GluN1-only spines increased with apoE3 and apoE4, but not with apoE2; and significantly decreased with apoE4 on DIV26.

Analysis of GluA2-only spines on DIV10 showed a comparable spine density across apoE isoform (Figure 4B). However, on DIV14, the highest density was with apoE3. On DIV18, the density of GluA2-only spines was comparable between apoE2 and apoE4, with a pronounced decrease with apoE3, perhaps due to acquisition of GluN1 by GluA2-only spines to form mature GluN1+GluA2-positive spines. On DIV21, a decrease in the density of GluA2-only spines was observed with apoE4, while no change was seen with apoE2 or apoE3. On DIV26, GluA2-only spines decreased with apoE2 and apoE3, and the decrease with apoE4 on DIV21 continued.

Drebrin-only spines were analyzed to examine changes in the density of spines not yet enriched with GluN1 or GluA2, or that may have lost their GluN1 and GluA2 (Figure 4C). Drebrin-only spines increased from DIV10 to DIV18 to densities with no significant difference between the isoforms. From DIV18 to DIV21, drebrin-only spines significantly increased with apoE4. On DIV26, drebrin-only spines increased with apoE2 and apoE3, but not apoE4. These data reveal a later phase of spine development when a decrease in the density of GluN1+GluA2-positive spines is accompanied by an increase in the densities of GluN1-only and drebrin-only spines and a decrease in GluA2-only spines. This suggests that the loss of GluA2 precedes the GluN1 loss in spines. This GluA2 loss occurs earlier with apoE4 spines and may contribute to the early loss of mature GluN1+GluA2-positive spines.

## DISCUSSION

Several studies demonstrate that apoE isoform may differentially influence synapse density, structure and function, with negative effects reported in the presence of apoE4 (Bour et al., 2008; Dumanis et al., 2009, 2013). Results presented herein utilize a co-culture model with natively regulated secretion of human apoE by glia, the main endogenous source of apoE in the CNS. The simultaneous analysis of changes in dendritic spines and dendritic spine glutamate receptor composition demonstrate an apoE4-induced delay in spine formation in developing neurons, and with increasing time in culture, an early loss of mature spines, compared with apoE2 and apoE3. Thus, these data provide evidence for a cellular and molecular basis for age-dependent apoE4 effects on neuron function *in vitro*.

APOE4 is associated with poor memory and cognitive performance and increased risk for AD in older individuals (usually after 50 years of age) (Deane et al., 2008; Caselli et al., 2009). However, young APOE4 carriers exhibit enhanced cognitive performance (Bachmeier et al., 2013), academic achievement (Hawkes et al., 2012) and a higher IQ (Yu et al., 2000) than non-APOE4 carriers. Young APOE4 individuals also show brain oxygen utilization comparable to APOE2, and higher than APOE3 individuals (Trachtenberg et al., 2012). Analysis of developing spine subtypes at maximal spine density showed that with apoE4, the density of GluN1+GluA2 dendritic spines was comparable to apoE3, but the density of NMDAR-rich and AMPAR-poor (GluN1-only) spines was greater with apoE4 than apoE3. These NMDAR-rich and AMPAR-poor spines in developing neurons are characteristic of immature spines that can undergo stabilization for memory formation and storage (Bourne and Harris, 2007). Thus, these differences in spine composition may provide a structural and molecular basis for the observed dichotomy of beneficial effects in young individuals with APOE4 and the detrimental effects of APOE4 in older individuals.

The slow development of dendritic spines observed with apoE4 may also be important in the poor recovery from damage by other insults, such as traumatic brain injury (Teasdale et al., 1997; Chiang et al., 2003; Zhou et al., 2008) or HIV-dementia (Burt et al., 2008; Chang et al., 2011). The apoE isoform-specific effects on inflammation may also be affecting dendritic spines. Aging is associated with increased pro-inflammatory signaling, which has been shown to induce internalization or removal of AMPA receptors from synapses (Hara et al., 2012). Interestingly, *in vivo* lipopolysaccharide (LPS) endotoxin treatment in APOE-TR mice induces glial activation and pro-inflammatory cytokine release, accompanied by synaptic protein loss, with a greater response in APOE4-TR mice compared with APOE2- and APOE3-TR mice (Zhu et al., 2012). The role of apoE isoform-specific neuroinflammation in the observed early loss of AMPAR from spines is of future interest.

The observed negative effects of apoE4 and positive effects of apoE2 on spines are consistent with their effects on AD risk. With apoE4, maintenance of GluA2 at spines was impaired, while the greatest density of GluA2-positive spines was observed with apoE2. Early loss of GluA2 from spines has several negative functional implications. Loss of AMPAR from spines may result in conversion of functional synapses into silent, non-functional synapses (Lo and Erzurumlu, 2007). GluA2 also plays a critical role in maintaining the biophysical properties of AMPAR, including reduction of AMPAR calcium permeability (Jonas et al., 1994; Geiger et al., 1995; Washburn et al., 1997). A decrease in GluA2-positive AMPAR can result in glutamate-induced excitotoxicity, long-term depression and eventual loss of spines (Luthi et al., 1999; Van Damme et al., 2002; Hsieh et al., 2006; Medvedev et al., 2008; Bell et al., 2009).
